# The Comparison of Accuracy of Post Space Digital Impressions Made by Three Different Intraoral Scanners: An In Vitro Study

**DOI:** 10.3390/diagnostics14242893

**Published:** 2024-12-23

**Authors:** Abdullah A. Meshni, Saurabh Jain, Hanan Nasser Marie Osaysi, Khadijah Nasser Hezam, Samar Samir Gomaan Adlan

**Affiliations:** 1 Department of Prosthetic Dental Sciences, College of Dentistry, Jazan University, Jazan 45142, Saudi Arabia; 2 Intern Clinic, College of Dentistry, Jazan University, Jazan 45142, Saudi Arabia; hanan.476477@gmail.com (H.N.M.O.); khadijahezam.dent@hotmail.com (K.N.H.); 3 MINA Dental Clinic, Ash Shati, Jazan 82725, Saudi Arabia; s.s.s.j.a23@gmail.com

**Keywords:** post and core, post space, post space impression, intraoral scanner, digital impression, trueness, accuracy

## Abstract

Background and Objectives: The present study aims to assess and compare the accuracy of post-space impressions captured by three different intraoral scanners (IOS) using various canal diameters. Methods: Three extracted natural maxillary central incisors were selected and prepared for a 1 mm wide margin and a 3 mm ferrule. All steps required for the endodontic procedure were performed, and the post space was prepared using post drills. The post length was kept constant at 12 mm, whereas the width was varied (Group 1: 1.4 mm, Group 2: 1.6 mm, and Group 3: 1.8 mm). Three IOSs (Trios3, iTero2, and Medit i700) were used to acquire a digital impression of the prepared post space. Each tooth was scanned 10 times by each scanner. So, in the end, 90 digital images were recorded, and the STL files were stored. GC Pattern resin was used to fabricate resin post and core patterns, which were scanned using an extraoral scanner (EOS). The STL file obtained was used as the reference file. To evaluate the trueness of the tested IOSs, each three-dimensional scan from an IOS was superimposed on the reference scan with the help of the Medit Design software 2.1.4. The software generates color plots and gives numerical values as deviations in the Root mean square (RMS) for the variance between the two superimposed scans. The data collected was tabulated for statistical analysis. One Way ANOVA was used to test the significance difference between three different IOSs, followed by Bonferroni Post-hoc test pairwise test to identify the differences between every two different IOS. Statistical significance was set at *p* < 0.05. Results: The mean deviation for trueness in post space impression values recorded by the Medit i700 was highest among groups 1, 2, and 3 [0.825 (±0.071), 0.673 (±0.042) and 0.516 (±0.039), respectively], followed by iTero2 [0.738 (±0.081), 0.569 (±0.043) and 0.470 (±0.037), respectively] and Trios3 [0.714 (±0.062), 0.530 (±0.040) and 0.418 (±0.024), respectively]. Significant differences were found between the groups for all three IOSs (Trios3: *p*-value < 0.0001; iTero2: *p*-value < 0.0001; Medit i700: *p*-value < 0.0001). Conclusions: Within the limitations of this study, it can be concluded that Trios3 IOS has higher accuracy (as it exhibited minimal deviation for trueness) in recording post space, followed by iTero2 and Mediti700 IOS. As the diameter of the post space is increased, the accuracy of recording by IOS increases. For all the tested IOSs (except for Trios3 and iTero2, when used to record post space with 1.8 mm canal diameter), the deviations in trueness were higher than the clinically acceptable limits. Thus, IOSs should be used cautiously when recording impressions of post spaces.

## 1. Introduction

The decision to preserve a tooth with significant loss of its coronal portion involves a careful evaluation of a variety of key factors [1]. Typically, these teeth need a post-and-core restoration, where the radicular portion provides support for the coronal component [2,3]. This coronal portion serves as the foundation for the retainer of a fixed dental prosthesis, which can be cemented or luted in place. Post-and-core restorations have a high success rate and are commonly used to treat severely damaged teeth [2].

Various materials and techniques are available for fabricating posts and cores, and both prefabricated and custom-made options are commonly utilized. Prefabricated posts and cores can be either metallic (such as brass) or non-metallic (such as glass fiber) [3,4]. They are popular due to their ease of use and the reduced time required during clinical procedures. Glass fiber posts, in particular, have a comparable modulus of elasticity, which helps prevent root fractures and provides good aesthetics, especially when used in the anterior region under all-ceramic crowns [5,6]. However, prefabricated posts also have some drawbacks, including inadequate bonding to the core [7] and poor adaptability to the root canal. This leads to uneven high cement thickness, causing microleakage and dislodgment due to debonding [8,9,10].

Custom posts and cores have historically been made from metal and are produced by casting after accurately recording the canal anatomy with impression materials, inlay wax, or pattern resin. These custom units provide excellent adaptability to canal morphology [11], and because they are cast as a single piece, there is no risk of separation between the post and core [12,13]. Nonetheless, a major drawback of custom-cast posts and cores is their high modulus of elasticity [2,14], which often leads to root fractures [15]. Furthermore, the fabrication process is highly technique-sensitive and demands significant clinical chairside time to capture the post-space anatomy accurately. Consequently, many dentists choose to use pre-fabricated posts and cores instead of custom options, given the inherent challenges associated with their use [16,17].

The digitalization of dentistry has significantly improved the quality of treatment provided to patients. It has also enabled dentists to work more efficiently with new digital technologies, saving time and effort. Digital impressions taken with intraoral and extra-oral scanners have demonstrated reliable results. Studies have shown that these scanners are highly accurate for recording impressions needed to fabricate indirect prostheses [18,19]. With digital technology, it is now possible to fabricate custom-made glass fiber and zirconia post and cores, thus reducing the drawbacks inherent with the use of metal cast post and cores [20,21,22].

Digital impressions of post space can be captured in two primary ways. The first method involves using conventional silicone impression or pattern resin to record the canal anatomy, which can then be digitized using an intraoral or extra-oral scanner [23]. Studies have reported that this technique is highly accurate. However, it has some limitations, such as requiring more chairside time and depending on patient compliance. The second technique utilizes intraoral scanners to capture the canal anatomy inside the patient’s mouth directly. This can be performed with the help of scan posts or without them [3,24]. Digital impression-making procedures have become common, resulting in a variety of brands of intraoral scanners (IOSs) available in the market [25,26]. In today’s competitive landscape, manufacturers continuously innovate and update their IOSs to deliver the best possible patient care. These innovations can be related to hardware developments, including introducing wireless devices, advancements in autocalibration, integrated heaters, and haptic feedback technology to guide image capturing [27,28,29]. Software innovations include the availability of an open interface, the incorporation of tailored software applications for treatment planning and simulation, and software enhancements to minimize image artifacts and omit superfluous imaging data [27,28].

Today, the use of IOSs extends beyond just image acquisition; they have applications in various areas of dentistry. These include three-dimensional tooth segmentation, placement of landmarks, model analysis, caries detection, monitoring tooth wear, evaluating soft tissues, assessing oral hygiene, and determining tooth shade [28,29].

The scanners from different companies operate on varying principles. Active wavefront sampling technology creates precise three-dimensional simulations by projecting optical rays in varying configurations and later recording the distortion of optical beam configuration [30]. In confocal technology, the laser beam reflected is selectively filtered from a pinhole aperture. This improves the contrast and resolution of the seized image [30]. The triangulation theory of image processing of the laser beam is the basis of IOSs using the triangulation technique [30]. In IOSs based on Optical coherence tomography (OCT), scans are collected by interferometry and a low-coherence light source [30]. Many companies utilize two or more technological principles to improve accuracy when fabricating IOSs [31,32]. Opaquers (powders or sprays) were used in older variants of the IOSs to improve accuracy. The latest IOSs do not require these opaquers, thus improving patient compliance [33,34].

Intraoral scanners reduce the chairside time associated with making impressions and lower material costs, making them indispensable in modern dental practice. The digital impression files can be easily transferred to production labs, eliminating the cumbersome process of transporting physical impressions [25,26,35,36]. Moreover, this technique is patient-friendly, making it suitable for challenging patients, including those who are uncooperative, have a high gag reflex, or have limited mouth opening. Dentists can immediately evaluate impressions and can rescan or modify them without wasting much time [25,26,32,35,36]. Applying IOS technology for bite registration, dynamic mandibular movement registration, full- arch implant impression, and post- space impression holds substantial potential. Limitations due to higher error rate hinder their use in these clinical procedures [37,38].

Research has demonstrated the high accuracy of intraoral scanners (IOS) when capturing digital impressions for tooth-supported and implant-supported short-span fixed and removable dental prostheses [39,40,41]. However, there are concerns regarding their effectiveness in recording impressions of post spaces. These scanners operate on the principle of capturing reflected light, but the limitation with post spaces arises from the inability of the light beam to penetrate to an adequate depth, making it challenging to obtain accurate recordings.

Few studies have evaluated the performance of different IOSs in capturing post-space impressions, and the findings have been inconsistent. Pinto et al. [22] compared the quality of impressions of post space at depths of 8.8 mm and 9.5 mm using intraoral scanners (IOSs) and conventional silicone impression techniques. They reported a significant discrepancy in the results from the IOS technique. Similarly, Hendi et al. [3] assessed the retention of posts and cores manufactured using conventional and digital impression methods, finding that conventional impressions resulted in higher retention for the posts and cores. Kanduti et al. [42] noted that most impressions showed greater discrepancies in the apical area compared to the cervical area when comparing digital and conventional impressions. Elter et al. [43] evaluated the accuracy of various IOSs in recording the depths of carious post space, reporting that accuracy decreases as the depth of the post space increases from 10 mm to 20 mm. The studies discussed used different types of IOSs, and there is no consensus on which scanner is superior for recording post space. The diameter of the prepared post space may influence the quality of impressions produced by the IOS. However, there is limited research on how canal diameter affects impression accuracy.

The present study aims to assess and compare the accuracy of post-space impressions captured by three different intraoral scanners using various canal diameters. The null hypotheses being tested are: 1. There will be no difference in the accuracy of post-space impressions obtained using different intraoral scanners, and 2. The width of the post-space canal will not affect the accuracy of post-space impressions recorded by different intraoral scanners.

## 2. Materials and Methods

The present study was approved by the Standing Committee for Scientific Research, Jazan University (Ref. No: REC-45/05/878). Non-carious natural maxillary central incisors, which were extracted for periodontal reasons, were used in the present study. All the selected teeth have approximately similar crown and root lengths (as evaluated by x-rays). A trained operator removed the anatomic crown of the teeth, 3 mm above the cemento-enamel junction, using a double-sided diamond disc to simulate a clinical scenario necessitating prosthetic rehabilitation by custom post and core [42,43]. The tooth was prepared to have a 1 mm wide margin and a 3 mm ferrule using a round-end tape diamond bur [42,43]. Details of all the materials and instruments used in the study are mentioned in Table 1.

**Figure 1:**
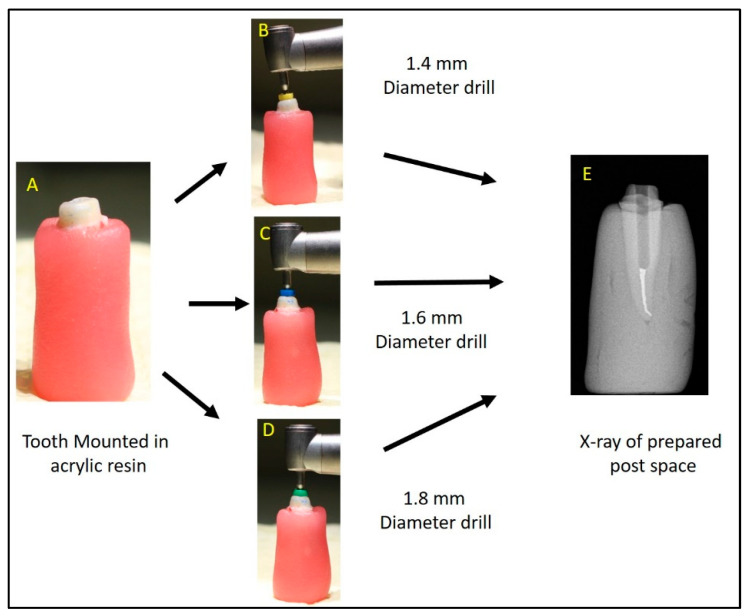
(**A**). Tooth mounted in resin; (**B**–**D**): Post space prepared using different dimensions of drills; (**E**): X-ray of tooth with prepared post space.

All the teeth were embedded in auto-polymerizing acrylic resin at the level of the cemento enamel junction. A trained operator performed all steps required for the endodontic procedure (pulp extirpation, BMP, Obturation) using a standardized protocol and rotary instruments (Protaper Gold, Dentsply Maillefer, Ballaigues, Switzerland) [44,45]. Post space was prepared using post drills (Relyx Fiber Post drills, 3M ESPE, Neuss, Germany) as per the recommendations provided by the manufacturer [44,45]. The post length was kept constant at 12 mm, whereas the width was varied, with group 1 having 1.4 mm, group 2 having 1.6 mm, and group 3 having 1.8 mm (Figure 1).

### 2.1. Sample Size Determination

The sample size was determined using the G*Power software (version 3.1.9.7, 2020; Heinrich Heine University, Düsseldorf, Germany). An F-tests within ANOVA was used to calculate the sample size. Taking references from published literature [43,46], the alpha error value was kept at 5%, the power of study at 85%, and the effect size (f) at 40%, the sample size of 10 per group was found to be suitable. To compensate for any faults in recording the impressions, one extra scan was performed per group.

### 2.2. Digital Acquisition of the Post Space Using IOS

**Figure 2:**
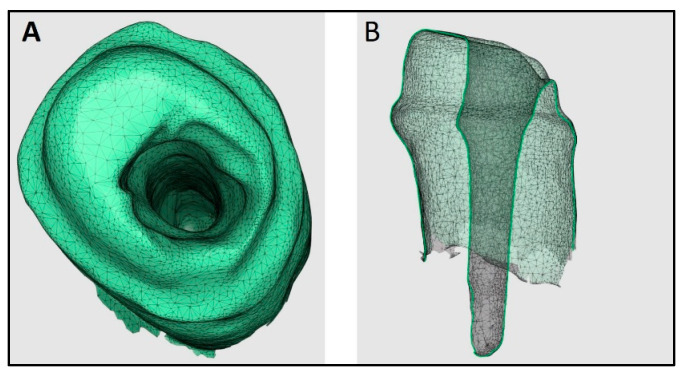
Digital impression made using Trios3 intra oral scanner. (**A**): Occlusal view, (**B**): Cross-sectional view.

**Figure 3:**
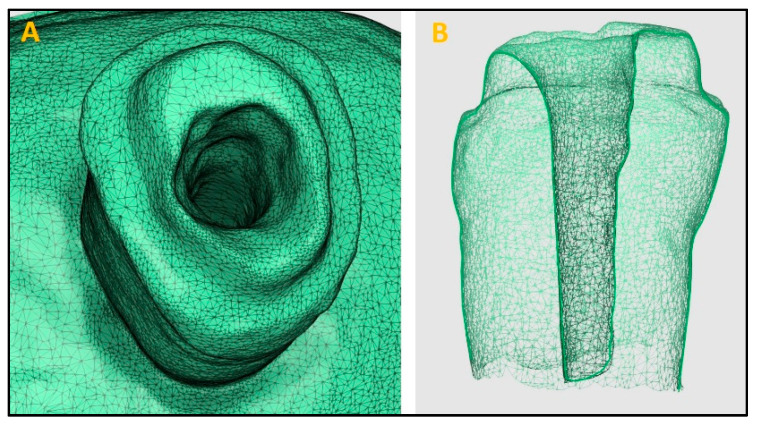
Digital impression made using iTero2 intra oral scanner. (**A**): Occlusal view, (**B**): Cross-sectional view.

**Figure 4:**
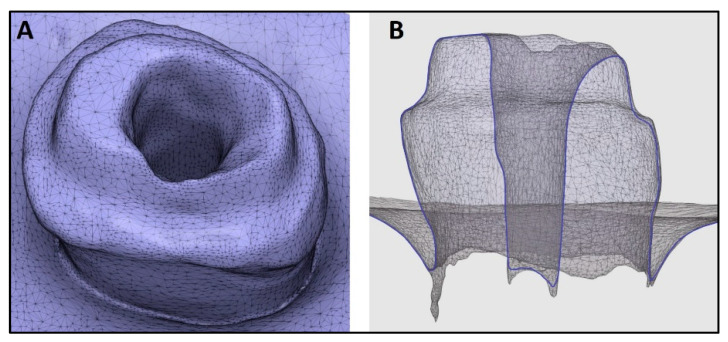
Digital impression made using Mediti700 intra oral scanner. (**A**): Occlusal view, (**B**): Cross-sectional view.

Three IOSs (Trios3, iTero2, and Medit i700) were used by a trained operator to acquire digital impressions of the prepared post space (Figure 2, Figure 3 and Figure 4). A standardized recroding protocol was used [43]. Confocal microscopy technology underpins the operation of Primescan and Itero2 IOSs, whereas the Medit IOS operates using the triangulation principle. [47,48]. Each tooth was scanned 10 times by each scanner. A gap of minutes was given between the scans to prevent scanner overheating and operator fatigue. So, at the end, 90 digital images (10 per scanner, per tooth) were recorded, and the STL files were stored.

### 2.3. Acquisition of Post Space Impression Using Extra Oral Scanner as the Reference Group

**Figure 5:**
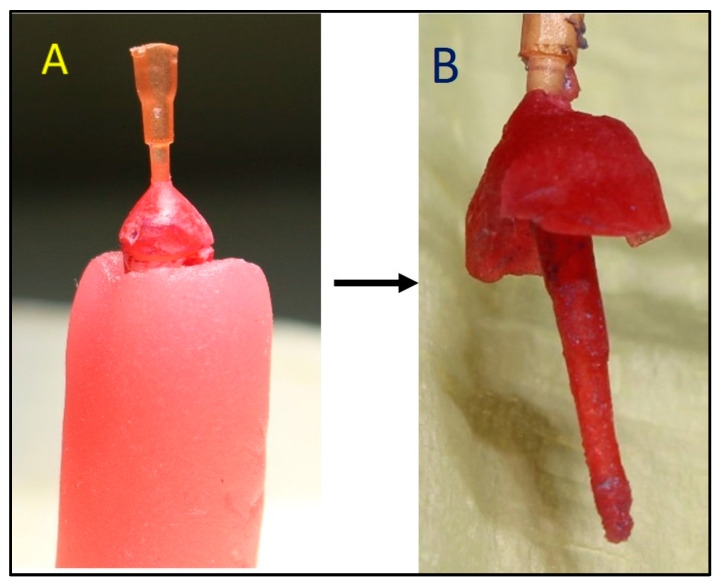
Pattern resin used to fabricate acrylic resin post and core pattern to record the anatomy. (**A**): Resin post and core pattern on the prepare tooth, (**B**): Resin post and core pattern after removal from the prepared tooth.

**Figure 6:**
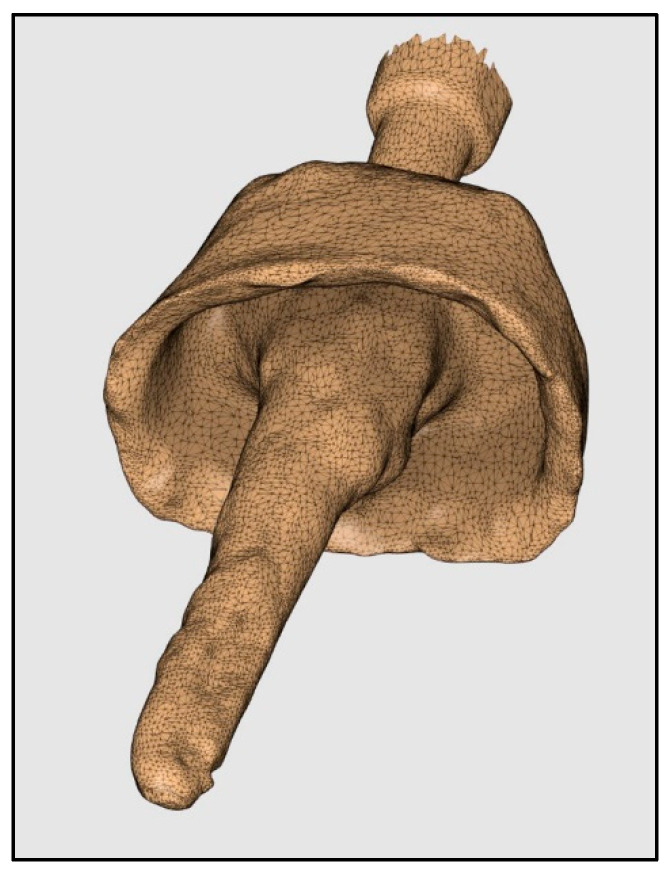
Resin post and core pattern scanned using extra oral scanner.

Pattern resin (Pattern Resin LS, GC America INC, Alsip, IL, USA) was used to fabricate acrylic resin post and core patterns to record the anatomy of the post space. Pattern resin was used with plastic post formers, and a standard protocol was used to record the canal anatomy (Figure 5). Later an extra oral scanner (3Shape E2 lab scanner, 3Shape, Copenhagen, Denmark) was used to scan these patterns, and the STL file obtained was used as the reference file to evaluate the accuracy of the IOSs (Figure 6).

### 2.4. Superimposition of STL Files

**Figure 7:**
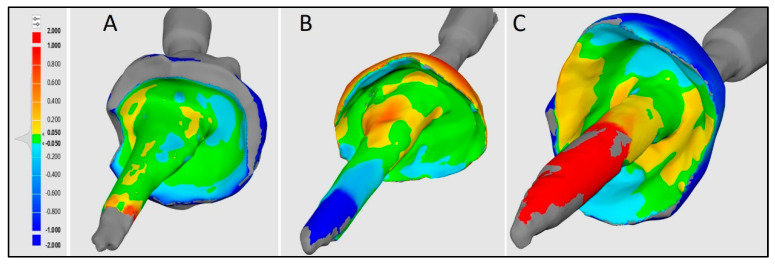
Color plot depicting 3-D deviation in trueness of intra oral scanners. (**A**): Trios3; (**B**): iTero2; (**C**): Mediti700.

The software used for this superimposition was Medit Design 2.1.4 (MEDIT Corp., Seoul, Republic of Korea) [49]. The observer that fed the STL files into the software was blinded and calibrated to perform the superimposition procedure (κ-value = 0.97). The accuracy of intra oral and extra oral scans was compared by superimposition using the Automatic alignment feature of the Medit link software. This software is used to align the data automatically without any user-defined points. Before evaluating trueness, the validation of the superimposition technique was necessary to ensure accurate recordings. One STL file was randomly selected, duplicated, and stored in a separate section. Now, these two duplicated files were superimposed multiple times to check the validity of the procedure. To evaluate the trueness of the tested IOSs, each three-dimensional scan from an IOS was superimposed on the reference scan obtained from the extra oral scanner with the help of the auto alignment element of the software. The reverse data feature of his software was used for aligning the two files. The software generates color plots and gives numerical values as deviations in the root mean square (RMS) for the variance between the two superimposed scans [50,51] (Figure 7). Higher RMS values denote lower trueness. Color codes generated can be read as Green representing the perfect alignment, Red-yellow denoting a positive/outward displacement, and blue-turquoise denoting a negative/inward displacement [33]. Following the previously mentioned protocol, all ten STL files from each scanner were superimposed, and the data collected was tabulated for statistical analysis.

### 2.5. Statistical Analysis

SPSS version 26 was used for data entry and analysis. Almost all the data were normally distributed by the Shapiro-Wilk test (*p*-value ≥ 0.05). However, the mean and standard division were calculated for all variables. One Way ANOVA was used to test the significance difference between the three groups, followed by a Scheffe post-hoc test for pairwise comparisons to identify the differences between every two groups. One Way Repeated Measure ANOVA was used to test the significance difference between three different intra-oral scanners, followed by Bonferroni post-hoc test pairwise comparison to identify the differences between every two different intra-oral scanners. Statistical significance was set at *p* < 0.05.

## 3. Results

The validation test reported minimal error (0.019 ± 0.004 μm) during superimposition, thus revealing that the methodology for trueness evaluation is reliable. Three groups of post space dimensions were scanned ten times each using three different intraoral scanners.

**Figure 8:**
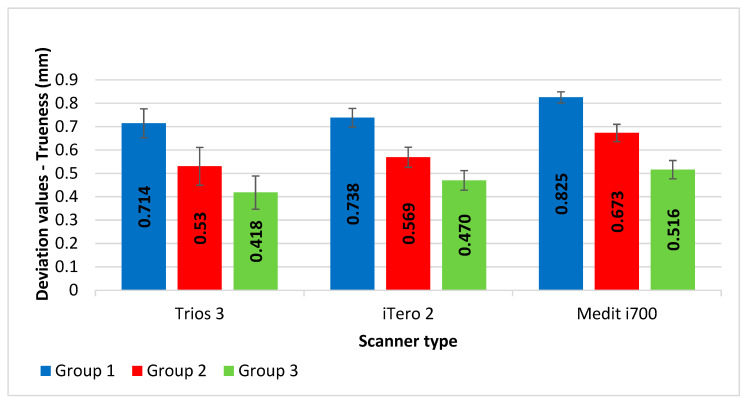
The mean deviation values for trueness of post-space impressions recorded by intra-oral scanners and groups.

Figure 8 shows the mean and standard deviation values for trueness for all three different intra-oral scanners (Trios3, iTero2, and Medit i700 scanners) across three groups. The mean (±SD) deviation for trueness in post space impression values recorded by the Medit i700 scanner was the highest among groups 1, 2, and 3 [0.825 (±0.071), 0.673 (±0.042) and 0.516 (±0.039), respectively], followed by iTero2 scanner [0.738 (±0.081), 0.569 (±0.043) and 0.470 (±0.037), respectively] and Trios3 scanner [0.714 (±0.062), 0.530 (±0.040) and 0.418 (±0.024), respectively].

Table 2 compares deviation values of trueness of post-space impressions recorded by three different intra-oral scanners (Trio3, iTero2, and Medit i700) for three groups. A one-way repeated measures ANOVA was performed to compare the accuracy of post-space impressions recorded by three different intra-oral scanners (Trios3, iTero2, and Medit i700) within each group. The results revealed significant differences in accuracy between the three scanners within each group (Group 1: *p*-value = 0.0023; Group 2: *p*-value < 0.0001; Group 3: *p*-value < 0.0001). A one-way ANOVA was conducted to compare the accuracy of post-space impressions between the three groups for each scanner. Significant differences were found between the groups for all three scanners (Trios3: *p*-value < 0.0001; iTero2: *p*-value < 0.0001; Medit i700: *p*-value < 0.0001).

Table 3 shows the results of Bonferroni post-hoc tests comparing the accuracy of post-space impressions between every two intra-oral scanners within each group. In group 1, statistically significant differences were found between Trios3 vs. Medit i700 (*p*-value = 0.0024) and iTero2 vs. Medit i700 (*p*-value = 0.0051), but not between Trios3 and iTero2 (*p*-value = 0.4930). Moreover, in groups 2 and 3, statistically significant differences were found between all pairwise comparisons: Trios3 vs. iTero2, Trios3 vs. Medit i700, and iTero2 vs. Medit i700 scanners [for group 2 (*p*-values = 0.0430, < 0.0001, and = 0.0002) and for group 3 (*p*-values = 0.0082, < 0.0001, and = 0.0146), respectively]. The largest differences in post-space impression values were found between Trios3 and Medit i700 scanners in all groups (0.111, 0.143, and 0.097 for groups 1, 2, and 3, respectively). The smallest differences were found between Trios3 and iTero2 scanners (0.024 and 0.039 for groups 1 and 2, respectively) and between iTero2 and Medit i700 scanners (0.045).

Table 4 shows the results of Scheffe post-hoc tests comparing the accuracy of post-space impressions between every two groups within each intra-oral scanner. In all three different intra-oral scanners, statistically significant differences were found between Group 1 vs. Group 2, Group 1 vs. Group 3, and Group 2 vs. Group 3 within each scanner [for Trios3 (*p* values < 0.0001, 0.0001, and 0.0001, respectively), for iTero2 (*p* values < 0.0001, 0.0001, and = 0.0025), respectively), and for Medit i700 (*p* values < 0.0001, 0.0001, and 0.0001), respectively). The largest differences in post-space impression values were found between Groups 1 and 3 in all three different intra-oral scanners (0.296, 0.268, and 0.310 for Trios3, iTero2, and Medit i700 scanners, respectively). The smallest differences were found between Groups 2 and 3 for Trios3 and iTero2 scanners (0.111 and 0.099, respectively) and between Groups 1 and 2 for Medit i700 scanners (0.152).

## 4. Discussion

This research involved using three different IOSs to make digital impressions of post space with three different canal diameters. When three IOSs were compared for trueness, minimal deviations were reported with the group using the Trios3 IOS, followed by iTero2 IOS. Maximum deviations were reported when Mediti700 IOS was used. Additionally, as the width of the post-space canal is increased, the deviation errors are reduced with each IOS. The results revealed significant differences in accuracy between the three scanners within each group and between the groups for all three IOSs. Therefore, both the tested null hypotheses were rejected. The magnitude of the differences in trueness varied with each IOS and the canal diameter.

IOSs have reported high accuracy in recording impressions for tooth and implant-supported prostheses. In addition to high accuracy and ease of use, reassessing, storing, and transferring digital impressions to production laboratories have made IOSs popular among dental professionals. Manufacturing companies are constantly improving these IOSs to provide the best possible outcome.

When IOS is used for recording digital impressions of crowns, the deviation values less than 120 μm were considered clinically acceptable [52,53]. However, these values can vary for post and core as the cement thickness of 250–500 μm is clinically acceptable. So, this can be taken as a clinically acceptable range for post and cores [54,55].

The use of IOS in recording post-space impressions is still under debate, especially in the apical third of the post-space. Hendi et al. [3] compared the apical gap in the posts fabricated by conventional, full-digital, and half-digital techniques. They reported higher apical gaps with digital technique (full-digital: 0.29 mm; half-digital: 0.66 mm) than conventional technique (0.11 mm). They reported that these apical gaps were within the acceptable clinical guidelines of 2 mm [11,56]. Kanduti et al. [42] compared the conventional and digital techniques and reported similar accuracy in the cervical part but significant differences in apical parts recording. The apical gap reported by the conventional technique was 53.66 ± 23.39 μm, whereas for the digital technique, this gap was reported to be 89.47 ± 19.30 μm. Leven et al. [57] compared two IOSs (Primescan and Trios 4) to record post space. They reported significantly higher linear discrepancies (lower accuracy) for impressions made by Primescan IOS compared to Trios4. The apical area reported lower accuracies for both the IOSs. Elter et al. [43] evaluated the trueness of one IOS (Primescan) when used to record different post-space lengths (10,12,14,16,18 and 20 mm). They reported that mean deviation values (in RMS) increased with the increase in the post-space length (357.1 μm for 10 mm post-space) to 897.5 μm for 20 mm post-space. They reported that Primescan IOS can be used to make digital impressions for the teeth with post space depth of less than 14 mm and a minimum diameter of 2.2 mm. Dupagne et al. [48] compared the accuracy of four IOSs (Primescan, Omnicam, TRIOS 4, and Medit i700) to record post space 10 mm deep. When the apical third of the post space is compared, the lowest errors (in μm) were re-ported by Trios4 (15.9 ± 1.3), followed by Medit i700 (16.4 ± 1.2), Omnicam (19.2± 3.2), whereas Primescan (19.5 ± 2) reported highest errors. Almalki et al. [44] used IOS (CEREC Primescan) to record post-space impressions of three different lengths (6, 8, and 10 mm). They reported higher apical accuracy for 6 mm post length (96 μm) and lower accuracy for 10 mm post space (163 μm). Non-significant differences in accuracy were reported for impressions in the coronal and middle third. They recommended using IOS for impressions in post space up to 8 mm in length. Emam et al. [45] used three IOSs (Primescan AC, Medit i500, and CS 3600) to record post space of 8 mm and 10 mm and reported higher deviations values (RMS) for CS 3600 (0.30 ±0.11 mm) and lowest values for Medit i500 (0.18 ±0.05 mm). Taha et al. [46] used three IOSs (Trios, Medit, and Primescan) and measured their trueness when used to record post space of two different cervical diameters (2.5 mm and 3 mm). For the 2.5 mm cervical diameter group, the highest deviations were reported by Trios (86.08 ± 2.50) followed by Medit (85.35 ± 5.46), whereas the lowest errors were reported by Primescan (36.21 ± 4.36). For the 3 mm cervical diameter group the highest errors were reported by Trios (39.55 ± 4.49), followed by Prime scan (38.27 ± 4.93), whereas the lowest errors were reported by Medit IOS (37.48 ± 10.37).

The outcomes of the reported studies support the limited use of these IOSs in recording post-space impressions. The present study used three different IOSs to capture digital impressions. The least deviations in trueness were reported with Trios3 (1.4 mm: 0.714 ± 0.062 mm, 1.6 mm: 0.530 ± 0.040 mm, 1.8 mm: 0.418 ± 0.024 mm), followed by iTero (1.4 mm: 0.738 ± 0.081 mm, 1.6 mm: 0.569 ± 0.043 mm, 1.8 mm: 0.470 ± 0.037 mm), whereas Medit i700 reported the maximum deviations in trueness (1.4 mm: 0.825 ± 0.071 mm, 1.6 mm: 0.673 ± 0.042 mm, 1.8 mm: 0.516 ± 0.039 mm). Studies have reported various factors that can affect the accuracy of these IOSs while making impressions. These factors can be machine-related (dimension of the tip of the scanner, sensitivity to color distinction, working principle of the scanner), operator-related (scanning procedure, skill, and training), and general environmental conditions related (light quality, temperature, and humidity). In the present research, a standardized recommended scanning protocol was followed by one trained and experienced operator to make all the digital scans. The difference in the deviations could be related to the machine-related factors as all three IOSs used have different dimensions of the scanning tip and work on different optical principles. Trios and iTero work on confocal microscopy, whereas Mediti700 works on the concept of triangulation.

A direct comparison of the results of our study cannot be made in other previous studies due to differences in methodology, the type of tooth in which the post space is measured, and commercial brands and generations of IOS used. The results of our study are in partial agreement with that of Taha et al. [46], who reported that an increase in the diameter of the post space increases the accuracy of IOS irrespective of the commercial brand. This could be explained by the fact that with an increase in the canal’s diameter, the IOS can project the optimal amount of light rays on the targeted area and capture back the reflected rays. The results of the present study are in agreement with Dupagne et al. [48] who reported lower deviation errors for trios IOS when compared to Medit IOS. In general, the results of the present study are not in full accordance with the results of studies by Taha et al. (for 3 mm cervical diameter) [46] and Emmam et al. [45], who reported lower deviations for Medit IOS when compared to Trios3, Primescan AC, and Trios4 IOS respectively. This could be attributed to various reasons. Firstly, it could be due to the use of different generations of IOS. In the present study, the generations of IOSs were used (Trios3, MEdi-ti700, iTero2), Whereas studies by Taha et al. [46] used Mediti600 Vs. Trios3 and Emmam et al. [45] used Mediti500 Vs. Trios4. Secondly, using different tooth models for the study can also contribute to these differences. Taha et al. [46] used single-rooted natural premolars, whereas Emmam et al. [45] used incisors and premolars. In the present study, natural maxillary central inci-sors were used. Additionally, differences can arise from variations in the size and shape of the prepared post spaces. Since the IOS operate on the principle of projecting optical rays that are subsequently absorbed, changes in the con-figurations of the canals can lead to variations in deviation errors.

The present study observed that as the diameter of the canal post space increased, the deviation errors decreased in each IOS. The highest deviations were noted with canal 1.4 mm, followed by 1.6 mm, and the least were noted with 1.8 mm. This could be explained by the fact that these IOSs work on the principle where the object reflects the optical rays sent by the IOS, and the scanner aperture/head reabsorbs these images to form a 3D image. The post canal’s anatomy inhibits the lights from getting reflected and recaptured correctly by the IOS. Thus, within the permissible range, the wider the canal will be, and the light beams can be projected, reflected, and absorbed better, leading to lower errors in recording. In the present study, only Trios3 and iTero IOS displayed deviations within the clinically acceptable range when making post space, which is 1.8 mm in diameter and 12 mm in length. All other tested parameters had higher values than the clinically acceptable range.

In the present study, scan posts are not used to record impressions of post space. Studies have reported discrepancies in the apical portion of the canal when scan posts are used [3,42]. This was attributed to the fact that scan posts come in different dimensions, and no available drill systems correspond to these dimensions, leading to the imperfect fit of these scan posts and eventually causing errors in recording post space [3,42]. Additionally, these scan posts are not FDA-approved [44].

In the present study, the software used for the superimposition of STL files and quantifying the deviation errors was Medit Design 2.1.4 (Medit Corp., South Korea), In contrast to Geomatic Control X (3-D systems) which was used in the research articles published before [43,45,46]. Medit Design is a non-meteorology grade software with an open system and accepts all STL files, whereas Geomatic Control X is a meteorology grade software. The use of Medit Design software in the present study is supported by the outcomes of the published literature, where it was reported that there is no significant difference in the accuracy assessment capabilities of these two software when used for the accuracy evaluation of crowns [49]. In the present study, all the canal shaping, obturation, and post-space preparation procedures were carried out using standardized recommended protocols by trained operators, thus minimizing the chances of human or technique-related errors.

Strengths and Limitations: The current study can be a vital addition to the pertaining literature due to the paucity of studies researching the accuracy of IOSs when used for post-space impressions with different diameters. The use of the commonly used generations of IOSs and vigorous and unbiased methodology are the key features of this research. Limitations include the in vitro nature of the study. This study does not consider various patient-related factors (lighting conditions, patient mouth opening, operator position) that can affect the study outcomes directly or indirectly. Also, the study is limited to only single-rooted maxillary anterior teeth, and the digital impressions were made only by three IOSs. Additionally, all the scans were made by the single operator, which could introduce bias. Thus, further studies involving more scanners and operators should be carried out, and the use of multi-rooted teeth should be considered in the future.

Clinical significance: This study guides dentists in selecting the right brand of IOS to make impressions of post space. It guides dentists on the appropriate clinical scenarios in which IOSs can be used without introducing errors in post-fabrication processes. However, it is not advisable to use IOSs for routine post-space impressions, particularly in cases where the post space is narrow or deep, as this may result in errors in the post and core restoration. The findings of this study could serve as a foundation for further advancements in digital technology.

## 5. Conclusions

Within the limitations of this study, it can be concluded that:

- Trios3 IOS has higher accuracy (as it exhibited minimal deviation for trueness) in recording post space, followed by iTero2 and Mediti700 IOS.
- As the diameter of the post space is increased, the accuracy of recording by IOS increases.
- For all the tested IOSs (except for the use of Trios3 IOS and iTero IOS, in post space with 1.8 mm canal diameter), the deviations in trueness were higher than the clinically acceptable limits. Thus, IOSs should be used cautiously when recording impressions of post spaces.

## Figures and Tables

**Table 1:** Details of Materials, Instruments, and Software Used in The Study.

Materials/Instrument	Company Details	Lot Number
Rotary Files	Protaper Gold, Dentsply Maillefer, Ballaigues, Switzerland	1461889
Gutta-percha	DiaDent group International, Chungcheongbuk-do, Republic of Korea	010321
Post Drills	Relyx Fiber Post drills, 3M, ESPE, Neuss, Germany	372580
Pattern Resin	GC America INC, Alsip, IL, USA.	907231
3Shape E2 lab scanner	3Shape, Copenhagen, Denmark	(21)-1UB2048014B
Trios 3 Intra Oral Scanner	3Shape, Copenhagen, Denmark	1C1843S01861B
iTero Element™ 2 Intra Oral Scanner	Align Technology, Inc., Tempe, Arizona	BLX2019W45A645
MEDIT i700 wireless Intra Oral Scanner	MEDIT 2024 Intraoral Scanners and Dental SoftwareSeoul, Republic of Korea	BF2301100612
Software	Medit Link 3.3.2 and Medit design 2.1.4, Medit Corp., Seoul, Republic of Korea	-

**Table 2:** Comparison the deviation values of trueness of post-space impressions recorded by three different intra-oral scanners between and within groups.

Groups	Trios3 Mean (±SD)	iTero2 Mean (±SD)	Medit i700 Mean (±SD)	Mean Square	F	*p* Value ^a^
Group 1	0.714 (±0.062)	0.738 (±0.081)	0.825 (±0.071)	0.034	8.651	0.0023
Group 2	0.530 (±0.040)	0.569 (±0.043)	0.673 (±0.042)	0.055	39.778	<0.0001
Group 3	0.418 (±0.024)	0.470 (±0.037)	0.516 (±0.039)	0.024	22.888	<0.0001
Mean square	0.223	0.183	0.240			
F	111.503	55.831	86.359			
*p* value ^b^	<0.0001	<0.0001	<0.0001			

^a^: One-Way Repeated Measure ANOVA; ^b^: One-Way ANOVA.

**Table 3:** Post-hoc test for comparison the accuracy of post-space impressions between every two different intra-oral scanners within each group.

Groups		Mean Difference	Std. Error	*p*-Value
Group 1	Trios3 vs. iTero2	0.024	0.03317	0.4930
	Trios3 vs. Medit i700	0.111	0.02669	0.0024
	iTero2 vs. Medit i700	0.088	0.02381	0.0051
Group 2	Trios3 vs. iTero2	0.039	0.01673	0.0430
	Trios3 vs. Medit i700	0.143	0.01520	<0.0001
	iTero vs. Medit i700	0.104	0.01783	0.0002
Group 3	Trios3 vs. iTero2	0.052	0.01538	0.0082
	Trios3 vs. Medit i700	0.097	0.01256	<0.0001
	iTero2 vs. Medit i700	0.045	0.01506	0.0146

Bonferroni Post-hoc test was used for pairwise comparisons between every two different intra-oral scanners.

**Table 4:** Post-hoc test for comparison the accuracy of post-space impressions between every two groups within each intra-oral scanner.

Scanner Type		Mean Difference	Std. Error	*p*-Value
Trios3	Group 1 vs. Group 2	0.184	0.019997	<0.0001
	Group 1 vs. Group 3	0.296	0.019997	<0.0001
	Group 2 vs. Group 3	0.111	0.019997	<0.0001
iTero2	Group 1 vs. Group 2	0.168	0.025594	<0.0001
	Group 1 vs. Group 3	0.268	0.025594	<0.0001
	Group 2 vs. Group 3	0.099	0.025594	0.0025
Medit i700	Group 1 vs. Group 2	0.152	0.023559	<0.0001
	Group 1 vs. Group 3	0.310	0.023559	<0.0001
	Group 2 vs. Group 3	0.158	0.023559	<0.0001

A Scheffe post-hoc test was used for pairwise comparisons between every two groups.

## Data Availability

The data that support the findings of this study are available from the corresponding author upon reasonable request.
